# Experimental Investigation of Polymer Injection in High Permeability Conduits for Material Sustainability and Behavior in Oil Reservoirs

**DOI:** 10.3390/polym15132950

**Published:** 2023-07-05

**Authors:** Sherif Fakher, Abdelaziz Lafi Khlaifat

**Affiliations:** Petroleum and Energy Engineering Department, School of Sciences and Engineering, American University in Cairo, New Cairo 11835, Egypt; sherif.fakher@aucegypt.edu

**Keywords:** polymer reinjection, material sustainability, polymer transport in porous media, polymer degradation

## Abstract

Polymers are one of the most widely used chemicals in the oil and gas industry. They are used for mobility control in enhanced oil recovery, in conformance control as a cross-linked plugging agent, as a fracking fluid for fracture propagation and proppant transportation, and in drilling fluids as an additive for drilling mud enhancement. This research characterizes the polymer injectivity in different pore sizes under different conditions and evaluates the polymer conditions after injection. Based on this, the ability to reinject the polymer in the porous media is discussed. The factors studied include the pore size, the polymer concentration, the polymer injection flowrate, and polymer injectivity. When the porous media size was reduced to 1.59 mm (1/16th of an inch), the injectivity value reduced significantly, reaching less than 0.2 mL/min/psi and the polymer degradation increased primarily due to shearing. Results also showed that the polymers underwent four main degradations during injection including dehydration, syneresis, shearing, and excessive hydrolysis. In continuous fractures, the degradation is a strong function of the fracture size, length, and the polymer structure. The experimental results showed that one or more of the polymer degradations resulted in the inability to reinject the polymer in most cases.

## 1. Introduction

Different chemical methods are used to enhance oil recovery based on the basic principles of improving sweep and displacement efficiencies. The chemical methods used are polymer-augmented waterflooding [1,2], surfactant flooding [3], alkaline flooding [4], CO_2_-augmented water flooding [5], and miscible/immiscible CO_2_ displacement [6]. Polymers have been utilized in the hydrocarbon industry for many years [1,2]. Their applications have also been expanding rapidly due to their superior qualities and wide range of selectivity. Regardless of the direction of their application in the oil and gas industry, one of the main ongoing challenges in polymers is their injection in the reservoir and propagation through the formation [7]. In order to inject the polymer, it undergoes very high shear rates and it also needs to be able to withstand very high pressure differentials and temperatures [8,9]. This usually results in severe degradation of the polymer structure, which leads to a reduction in operational efficiency and the inability to reuse the injected polymer for material sustainability [8,9]. One of the main methods by which this can be overcome is by characterizing the polymer and understanding its behavior under different conditions to evaluate the severity of polymer degradation. 

Many researchers have conducted core flooding and simulation experiments to investigate the ability of polymers to increase oil recovery and reduce polymer adsorption to different formation types. Core flooding experiments were aimed at understanding the behavior of polymer injection [10,11,12], multi-phase flow [12], the oil recovery potential [13,14], and the residual oil concentration after polymer injection [15,16,17,18]. Some core flooding experiments also focused on the ability of the polymer to displace extremely heavy oil, with viscosity values up to 25,000 cp [19]. Reservoir simulation studies utilized fine-grid simulation [20] and numerical simulation to examine large-scale polymer injection and the impact of several parameters, including cross-flow and heterogeneity [21,22,23,24,25]. 

Polymer injection has also been coupled with other EOR methods [26,27,28,29,30]. One of the most commonly used polymers is synthetic polymer, primarily HPAM [26]. In most of the research studies, polymer injection was combined with other chemical EOR methods, such as alkaline or surfactant flooding. When combining the polymer with alkaline, both the macroscopic and microscopic sweep efficiencies may be improved. The alkaline was used to react with the crude oil downhole and create an in situ surfactant, which can reduce the residual oil saturation, while the polymer was used as a mobility control agent [5]. Surfactant flooding was also combined with polymer flooding to reduce surfactant adsorption to the reservoir by sacrificing the polymer [31]. Researchers have also investigated polymer flooding with low-salinity water flooding and thermal injection methods [5,32,33,34].

Polymer is also used in conformance control processes and in hydraulic fracturing as a cross-linked polymer. There are more than 100 different types of cross-linked polymers present. These vary in the type of polymer used and the type of cross-linker. They are generally classified as organic or inorganic depending on the origin of the chemical. The selection of the polymer and cross-linker is based on the reservoir fluid properties, such as total acid number, oil viscosity, formation water pH and salinity, and concentration of monovalent and divalent cations. The reservoir thermodynamic conditions also govern the selection of the chemical since many chemicals are limited in terms of temperature and pressure conditions [5,32,33,34,35,36]. 

Based on the aforementioned details, polymers are an extremely popular chemical in the oil and gas industry for many applications. Although much research has been conducted on HPAM injection and propagation, the idea of polymer reuse through reinjection has recently caught much attention. This research investigates the impact of different factors on polymer degradation. The research then links these degradations to the ability to reinject polymer in the porous media for polymer reuse. The research provides several recommendations for HPAM polymer to avoid degradation and for its potential reinjection into porous media, when used to enhance oil recovery, for material sustainability and cost reduction. 

## 2. Experimental Description

### 2.1. Experimental Materials Used

The experimental materials used to conduct all experiments in this research are as follows:

Acrylamide: The acrylamide monomer, used to synthesize the polymer, was purchased from Sigma-Aldrich with a MW of 71.08 g/mol as a white powder with 99.99% purity.

Initiator: The initiator used to polymerize the acrylamide was potassium persulfate (K_2_S_2_O_8_). It was purchased from Sigma-Aldrich as a white powder with 99.99% purity.

Argon: Argon was provided as a high-pressure cylinder with 99.99% purity.

High-Temperature Oven: The oven was used to partially hydrolyze the polymer. The oven’s maximum temperature could reach up to 523 K (250 °C). The degree of hydrolysis of the polymer ranged between 10 and 15%.

Distilled Water: Distilled water was used to displace the fluids during the experiments and also to create the polymer solutions with different concentrations. 

Pressure Transducers: High-accuracy pressure transducers were used to record the pressure values during polymer injection. The transducers recorded one pressure reading every second. 

Magnetic Stirrer: The magnetic stirrer was used to mix the polymer powder with the distilled water and to ensure that the mixture was homogenous. 

Stainless Steel Tubes: High-pressure stainless steel tubes were used to mimic the small-diameter conduits that represent porous media. The tubes had an ID of 1.59 mm (1/16 inch) and 3.175 mm (1/8 inch). The stainless steel tubes had a high internal roughness induced through the injection of abrasive material to mimic the roughness of the porous media. 

### 2.2. Polymer Synthesis

**Figure 1:**
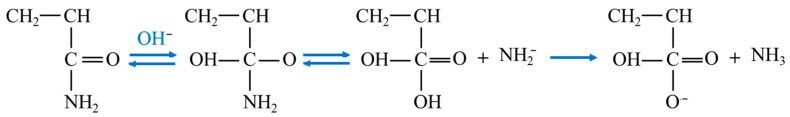
HPAM polymer synthesis.

In order to synthesize the hydrolyzed polyacrylamide (HPAM), the monomer acrylamide is used. The polymerization process is illustrated in Figure 1 The polymerization reaction is necessary to create long chains of the monomer. The reaction involves the addition of an initiator, potassium persulfate (KPS), which generates the required radical that adds to the double bond of another acrylamide, thus initiating polymerization and creating polyacrylamide (PAM). The weight percentage was 77% water, 22.2% monomer, and 0.8% initiator. During polymerization, an inert gas (Nitrogen or Argon) is introduced into the solution to purge any oxygen presence to avoid significant damage to the structure of the polymer by oxygen. Although the PAM can be used in this phase as a polymer, hydrolyzing the PAM to create HPAM is much more preferable since it introduces the carboxylate group which improves the performance of the polymer greatly. Hydrolysis is carried out through different methods depending on the polymer used. In this case, the PAM was partially hydrolyzed by placing it in an oven at 40 °C for a short duration, less than two hours. Placing the polymer at a higher temperature or for a longer duration could result in excessive hydrolysis which leads to severe degradation of the polymer structure, which in turn will have a negative impact. After polymerization, the average molecular weight of the HPAM was determined to be 5,500,000 g/mol.

### 2.3. Experimental Setup

The setup is composed of a syringe pump used to inject distilled water and to displace the polymer through the accumulator. The accumulator houses the polymer and is directly connected to the tube, with a valve at the outlet of the accumulator to control the polymer flow. The tube has a length of three feet with two pressure transducers located equidistant from each to record the pressure at the inlet of the tube and at the middle of the tube. The tube was maintained at the same level as the accumulator and was maintained in a horizontal position to remove the effect of gravity, which is not the focus of this study.

### 2.4. Experimental Procedure

All experiments were conducted using the same procedure in order to be able to accurately compare the results. The experimental procedure is as follows:

- Synthesis of the polymer using the polymer synthesis procedure discussed above and then hydrolyze the polymer by placing it in the oven until it reaches 10–15% hydrolysis. Dehydrate the polymer until it becomes a dry white powder.
- Prepare the polymer solution by placing the polymer powder in the distilled water and stirring the mixture for at least 12 h using a magnetic stirrer. The solution should be covered during stirring to avoid any contaminants entering the solution during the mixing process.
- Place the polymer in the accumulator and ensure that there are no air pockets between the polymer which may impact the experiments. Also, the accumulator must be fully occupied by the polymer.
- Connect the tube to the accumulator and attach the pressure transducers after they have been calibrated.
- Begin the polymer injection using the lowest flowrate. Each flowrate is stopped when the pressure becomes stable for at least two pore volumes of injection.
- Once the experiment is concluded, the accumulator and tube are cleaned and prepared for the following experiments.

## 3. Results and Discussion

The results, obtained from this research and discussion in this section, include the polymer injection plots at different flowrates for the different polymer concentrations; the average injection pressure for every flowrate; the injectivity of the polymer at different flowrates; the impact of polymer concentration on injectivity; and the polymer degradation mechanisms and the viability of polymer reinjection. The tubes used had a uniform ID to mimic small non-continuous fractures and high permeability features occurring near the wellbore. For more complex fracture networks, a more complex model is needed.

### 3.1. Polymer Injection and Injectivity

**Figure 2:**
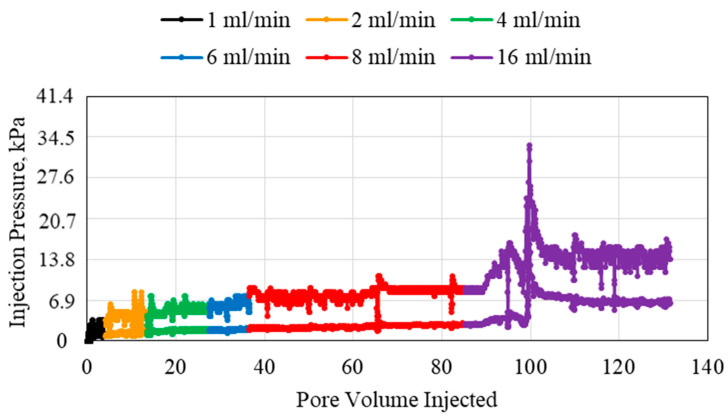
0.1 wt% HPAM injection at different flowrates using a 1.59 mm (1/16th inch) tube.

The results for the polymer injection using 0.1 wt% polymer, 1.59 mm (1/16th inch) tube, and 1, 2, 4, 6, 8, and 16 mL/min flowrates are shown in Figure 2. Increasing the injection flowrate resulted in two significant observations. A larger pore volume of the polymer was needed to ensure that pressure was stable when the flowrate was higher. This indicates that using a high flowrate may require a large volume of polymer. At the higher flowrates, 16 mL/min, more abnormalities in flow behavior were observed. For example, for the 16 mL/min injection flowrate, a sudden increase in injection pressure occurred. This is due to the dehydration of some of the injected polymer under higher pressure gradients. This resulted in the polymers becoming solid-like which makes their injectivity much more difficult. Also, increasing the flowrate will increase the overall volume of the polymer flowing through the porous media. This creates a competitive flow which results in an increase in pressure. Another major observation is that at higher flowrates, the polymer-injected pore volume needed for the injection pressure to stabilize was higher. This is due to the turbulence created at higher rates. 

**Figure 3:**
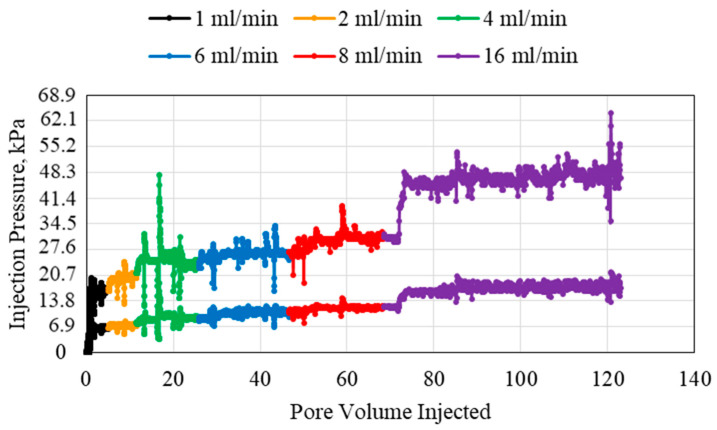
0.5 wt% HPAM injection at different flowrates using a 1.59 mm (1/16th inch) tube.

When the polymer concentration was increased from 0.1 wt% to 0.5 wt%, several observations were obtained. The injection pressure plot for the 0.5 wt% polymer is presented in Figure 3. The overall pressure increased with an increase in polymer concentration. However, it was observed that the pressure increase in the first pressure transducer, at the inlet, was higher compared to the second pressure transducer, at the middle, when comparing the 0.1 to the 0.5 wt% polymer. This indicates that the initial injection pressure of the polymer was affected more when the polymer concentration is increased. The abnormalities in flow were more pronounced with the increase in polymer concentration.

**Figure 4:**
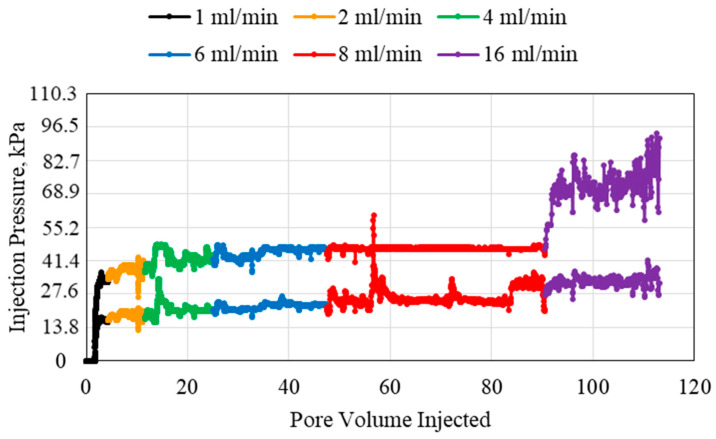
1 wt% HPAM injection at different flowrates using a 1.59 mm (1/16th inch) tube.

Figure 4 shows the pressure results when the polymer concentration was increased to 1 wt%. Overall, the pressure increased beyond that of the 0.5 wt% polymer. At the highest flowrate, 16 mL/min, the flow abnormalities were extremely high, especially for the inlet pressure. When the produced polymer was analyzed, it was found that the polymer produced at the end of the experiment had formed a coagulation which resulted in the sudden pressure increase seen at the final point in the 16 mL/min flowrate. This was not significant in the middle pressure reading due to the overall lower pressure values seen at that point in the tubes.

**Figure 5:**
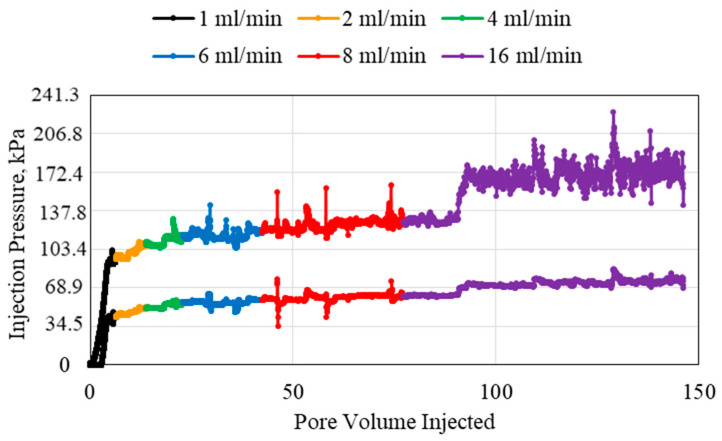
2 wt% HPAM injection at different flowrates using a 1.59 mm (1/16th inch) tube.

The highest polymer concentration used in this study was 2 wt% polymer, as shown in Figure 5. The pressures recorded for this polymer injection were the highest observed in all experiments. The main observation from the 2 wt% polymer experiment is that the highest flowrate required an extremely large injection pore volume in order to be able to decisively observe an average stable pressure. There are also several sudden increases in pressure for all the injection flowrates, which may indicate difficulty in injection or turbulence or slight air pockets in the polymer solution. 

**Figure 6:**
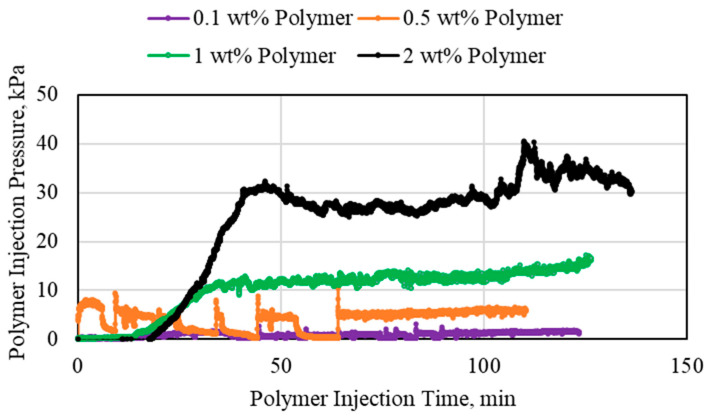
Polymer inlet section injection pressure for different polymer concentrations using 3.175 mm (1/8th inch) tubes.

**Figure 7:**
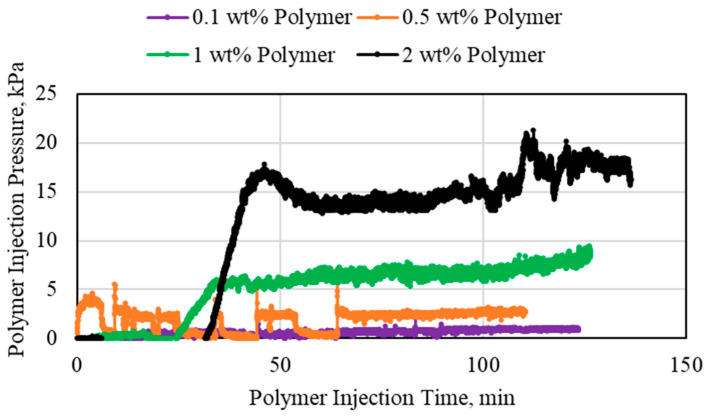
Polymer middle section injection pressure for different polymer concentrations using 3.175 mm (1/8th inch) tubes.

The porous media pore size was then doubled to 3.75 mm (1/8th) of an inch instead of 1.59 mm (1/16th) of an inch. This resulted in a significant decrease in the overall injection pressures due to the increase in the contact area of the porous media and the available cross-sectional area for the flow. The polymer injection pressures using flowrates 1, 2, 4, 6, 8, and 16 mL/min for all the polymer concentrations at the inlet and the middle are presented in Figure 6 and Figure 7, respectively. For the lower flowrates, i.e., less than 4 mL/min, and the low polymer concentrations, the pressure readings were very low and close in value to each other. This is mainly due to very little resistance to flow in the 1/8-inch tube, which resulted in pressure readings that were very close in value. As the injection flowrate increased, the pressure increased for all the polymer concentrations. This indicates that even though increasing flowrate may improve injectivity, it will also increase pressure requirements, which is something that must be considered in the polymer injection design. Overall, the higher polymer concentration resulted in a larger pressure due to the higher viscosity of the polymer solution. Some pressure abnormalities can also be seen in the plot, which are mainly due to the presence of small air pockets that could not be overcome. The air pockets resulted in a cessation of flow for a small period accompanied by a sudden increase in pressure and followed by a decrease in pressure. Overall, all the injection pressures are much lower than those of the 1/16th-inch tubes.

### 3.2. Polymer Average Stable Pressure

**Figure 8:**
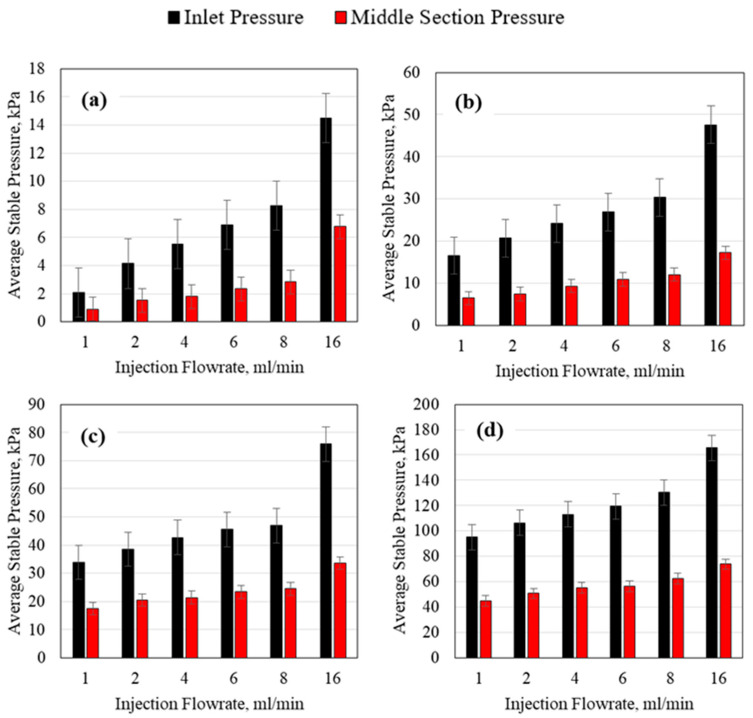
HPAM average stable injection pressure using a 1.59 mm (1/16th inch) tube ID for (**a**) 0.1 wt% HPAM, (**b**) 0.5 wt% HPAM, (**c**) 1 wt% HPAM, and (**d**) 2 wt% HPAM.

For all the polymer solutions injection, the average stable pressure obtained at each flowrate was recorded. The pressure was recorded as an average since small fluctuations were observed during all the experiments and thus an average value had to be taken. The average value was recorded when the pressure change was extremely low for at least two pore volumes of injection for each flowrate. The average stable pressures obtained for the 0.1, 0.5, 1, and 2 wt% polymer concentration at both the inlet and middle sections of the tube for the 1.59 mm (1/16th inch) tube ID are presented in Figure 8. Increasing the flowrate for all the polymer concentrations resulted in an increase in the average stable pressure. The increase was higher at the inlet which indicates that the length of the porous media may play a significant role in the injection pressure of the polymer, as will be explained later. As the polymer concentration increased, the average stable pressure also increased; however, it is noteworthy to mention that for the 2 wt% polymer, the increase in pressure was much more significant than any other polymer concentration. This indicates that at some point, the polymer concentration increase will impact the ability of the polymer to propagate through the small-sized porous media. This was also observed by Seright et al. [36] who showed that at some polymer concentrations, the polymer will not be able to propagate through the porous media. It is important therefore to consider the injectivity of the polymer and its ability to propagate through a specific pore size when choosing the polymer concentration design.

**Figure 9:**
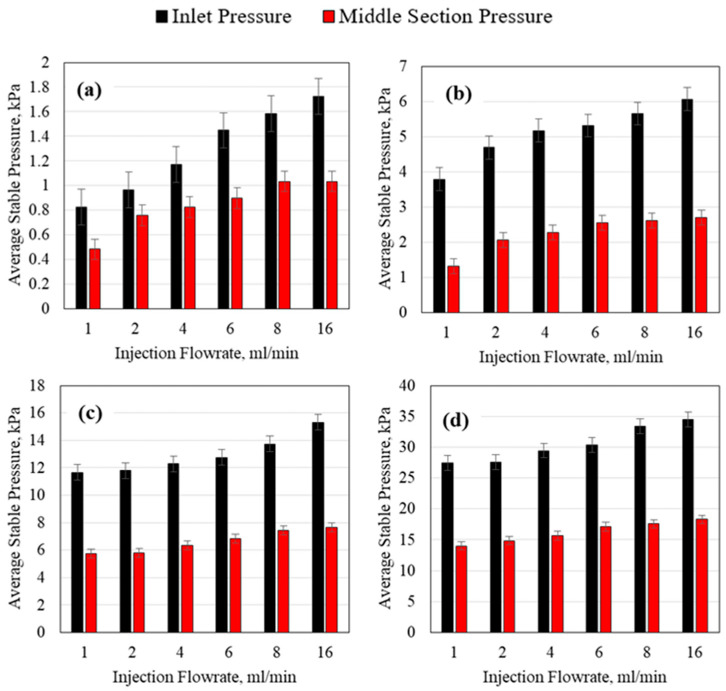
HPAM average stable injection pressure using a 3.175 mm (1/8th inch) tube ID for (**a**) 0.1 wt% HPAM, (**b**) 0.5 wt% HPAM, (**c**) 1 wt% HPAM, and (**d**) 2 wt% HPAM.

The average injection pressures obtained from the 1/8th-inch tubes for both the inlet and the middle sections are shown in Figure 9. An average pressure value was used because even during stable pressure, the pressure reading fluctuated slightly; thus, an average pressure for five continuous minutes of stable pressure was taken. For all experiments, the inlet pressure was much higher than the middle section pressure. This illustrates the impact of the porous media length. As the length of the porous media increases, a higher inlet pressure will be required to displace the polymer along the formation. Increasing the polymer concentration resulted in a non-linear increase in the pressure requirement as well. The injection flowrate was also found to impact the stable pressure non-linearly. Based on these results, it is apparent that even though a high polymer concentration may be favorable for increasing oil recovery, the polymer may be very difficult to inject which may result in the failure of the injection operation.

### 3.3. Polymer Injectivity

Injectivity is defined as the ability of a fluid to be injected into a conduit or porous media. It is the ratio between the injection flowrate and the pressure observed at the inlet of the conduit during the injection of a fluid into the conduit. As the injectivity value increases, the ability to inject a specific fluid also increases; therefore, during polymer injection operations, a high injectivity value is favorable. 

**Figure 10:**
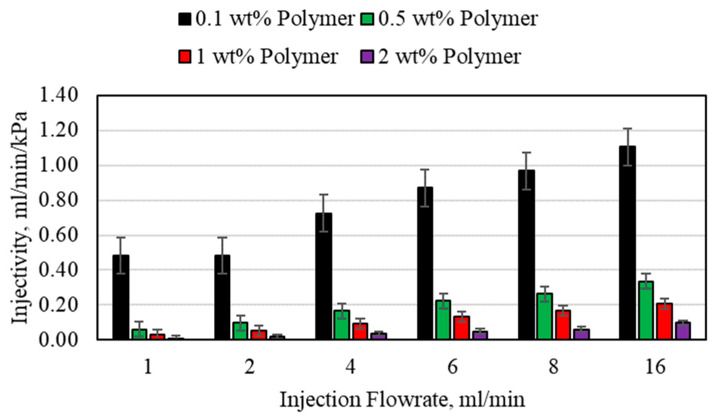
Polymer injectivity for the 1.59 mm (1/16th inch) porous media.

The lower polymer concentration, including 0.1 and 0.5 wt%, exhibited a good injectivity compared to the higher polymer concentrations for both the 1/8th- and 1/16th-inch tubes. The injectivity values for the 1/16th-inch tubes are presented in Figure 10 for all polymer concentrations. For the 0.1 wt% polymer, the injectivity was higher than unity for all the flowrates, which is an indication of a very favorable injectivity for this polymer concentration. When the concentration increased to 0.5 wt%, it is observed that at the lower injection flowrates, less than 4 mL/min, the injectivity falls lower than unity. Thus, the injection flowrate requirement increased with the increase in the polymer concentration. The overall injectivity for the 0.5 wt% polymer was still much better compared to the 1 and 2 wt% polymer concentrations. When the polymer concentration increased beyond 0.5 wt%, the injectivity value began to fall beneath unity even at high flowrates, which is considered extremely unfavorable. For the 1 wt% polymer, the injectivity remained beneath unity until the flowrate increased beyond 6 mL/min. This shows that as the polymer concentration increases, a higher injection flowrate becomes extremely important. This is also clear from the 2 wt% injectivity results, where even the 16 mL/min flowrate could not increase injectivity beyond unity. A higher injection flowrate will require higher strength surface pumps and also may cause formation damage if the formation fracture pressure is exceeded. Thus, a high polymer concentration requires extensive design precautions before implementing it in a field study. 

**Figure 11:**
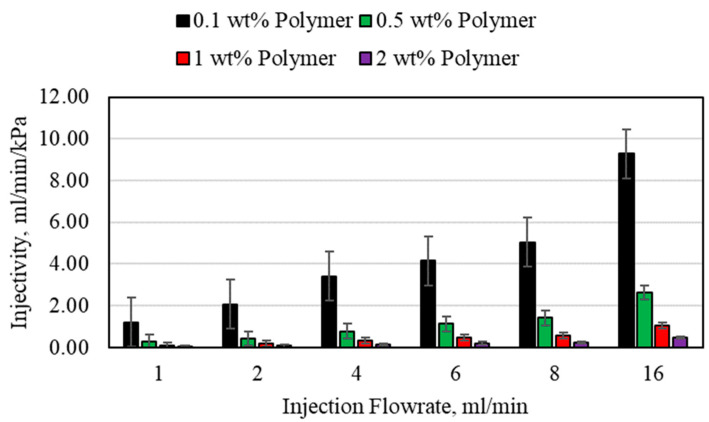
Polymer injectivity for the 3.175 mm (1/8th inch) porous media.

The injectivity results for the HPAM polymer using different polymer concentrations and different injection flowrates in the 3.175 mm (1/8th inch) tube are shown in Figure 11. Increasing the injection flowrate for all the polymers resulted in an increase in injectivity. The main problem with increasing injection flowrate is the increase in pressure as well, as was shown previously. If the pressure exceeds the formation fracture pressure, complications may arise downhole. As the polymer concentration increases, the injectivity decreased significantly as well. For example, the 2 wt% polymer injectivity could not exceed 3.2 mL/min/psi, which is lower than the lowest value obtained using the 0.1 wt% polymer, 8.33 mL/min/psi. For all values, the injectivity in the larger-diameter porous media was much higher compared to the smaller porous media. This will also reflect on the polymer degradation and reinjection significantly.

**Figure 12:**
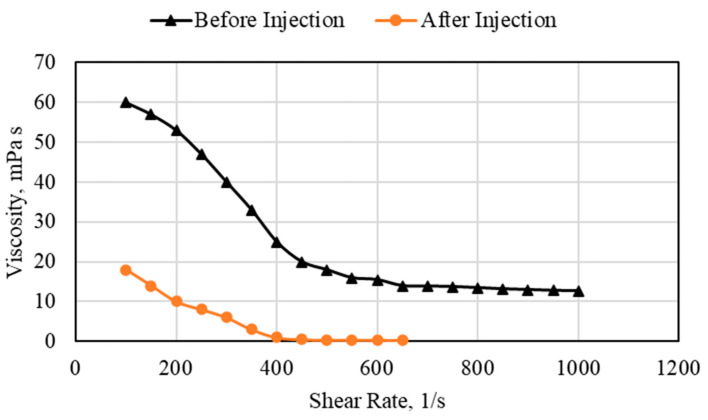
Polymer viscosity before and after injection using the 1 wt% polymer.

The polymer viscosity reduction due to injection for the 1 wt% polymer injected in the 1/16th-inch tube is presented in Figure 12. For both cases, the polymer viscosity reduced with the increase in the shear rate. This was due to the degradation of the polymer chain at high shear rates. For the sample before injection, the polymer viscosity reached 16 cp at the highest shear rate used, 1200 1/s. For the polymer sample after injection, the sample has a much lower initial viscosity due to degradation during injection. The polymer viscosity reached less than 1 mPa s at a shear rate beyond 400 1/s. This is a direct indication of polymer degradation during injection. This also gives rise to the importance of understanding the mechanisms by which the polymer can degrade. 

### 3.4. Polymer Degradation and Reinjection

**Figure 13:**
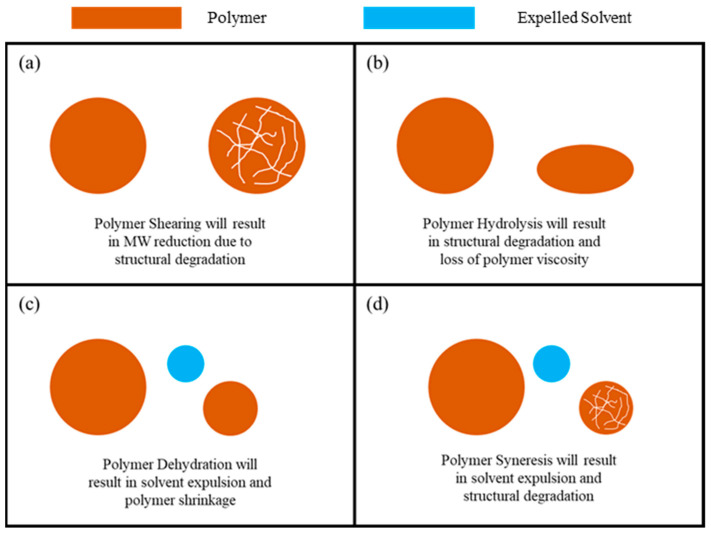
Polymer structural degradation mechanisms: (**a**) shearing, (**b**) hydrolysis, (**c**) dehydration, and (**d**) syneresis.

During its injection and propagation through the porous media, the polymer was subjected to severe thermodynamic and operational conditions, which resulted in polymer degradation. Degradation in polymer structure can be divided into four main types, illustrated in Figure 13, including:

Polymer Shearing: Polymer shearing refers to the reduction in the molecular weight (MW) of the polymer due to mechanical forces. These can include high pressure gradients and shearing in the surface equipment during injection. Polymer shearing is an irreversible process which will cause permanent degradation of the polymer structure. It results in a significant reduction in the polymer viscosity, which is a paramount functional property of the polymer.

Polymer Hydrolysis: Polymer hydrolysis is the controlled breakdown of the polymer structure using chemical reactions or thermodynamic conditions. Although partial hydrolysis is beneficial, as was explained previously, uncontrolled hydrolysis can result in severe polymer degradation, MW reduction, and loss of viscosity. Uncontrolled hydrolysis can occur in the formation due to two main impacts: the first is the presence of chemicals that can degrade the polymer structure such as acids and the second is the reservoir temperature, which may cause hydrolysis in some polymers based on their thermal stability.

Polymer Dehydration: Polymer dehydration is the expulsion of the solvent, usually the water, from the polymer due to high pressure gradients. This results in a significant increase in the polymer viscosity which in turn causes fluctuations in the injection pressure, as was observed in many of the injection experiments in this research. Polymer dehydration can be reversed by rehydrating the polymer using the design solvent as long as the polymer structure was not affected. The main contributor to dehydration is the high-pressure differentials that occur during the injection.

Polymer Syneresis: Polymer syneresis is the structural degradation of the polymer due to the expulsion of solvent or change in polymer properties. Similar to polymer dehydration, during syneresis, the viscosity of the polymer is expected to increase. Unlike dehydration, however, syneresis is an irreversible process due to structural degradation of the polymer. Factors that contribute to syneresis include pressure differentials that impact the polymer structure, high temperatures that result in solvent loss, and chemical agents in the reservoir such as monovalent and divalent cations, oxygen, and other agents that result in solvent loss. 

For the 3.175 mm (1/16th inch) experiments, the above-discussed four degradation mechanisms were observed. Due to the small cross-sectional and surface areas of the porous media, the injection pressure increased. This increase was much more substantial when the polymer concentration increased. This resulted in shearing action during injection. Also, polymer dehydration and syneresis were observed during injection when abnormal pressure peaks occurred. This was due to the polymer losing water which resulted in a sudden increase in polymer viscosity. This was translated as an immediate increase in pressure. When the polymer was produced from the outlet, solid lumps were observed which supports the claim that dehydration occurred. An attempt was conducted to rehydrate the polymer; however, it was not successful. This is a good indication that the polymer structure was degraded, therefore syneresis occurred. Finally, slight polymer dehydration occurred due to an increase in temperature during injection; however, its impact was not substantial. 

When the porous media size was increased to 3.175 mm (1/8th inch), the degradation of the polymer decreased significantly. The polymer exhibited almost no dehydration or syneresis which is evident from the injection curves which show no extreme fluctuations in the pressure. The shearing and hydrolysis effects were minimal; this was determined by measuring the viscosity of the polymer samples before and after injection. It was found that a negligible change in viscosity occurred which means that the sample maintained its structural integrity after injection, propagation through the porous media, and production. 

Based on the experimental results and the different mechanisms of polymer degradation, there are multiple parameters that may impact the process of reinjecting the polymer. These parameters can be grouped into three broad categories: reservoir rock properties, reservoir fluid properties, and operational properties. The reservoir rock properties that will strongly impact polymer degradation based on this research are the pore size and pore-size distribution, permeability, and porous media length. The fluid properties include the interaction between the polymer and the oil, formation water, acidic components, and gasses (if present). Finally, the operational properties include the average reservoir temperature, injection pressure at the surface, and the expected pressure differential in the formation. As the porous media length increased, the pressure required to inject the polymer also increased. This resulted in polymer degradation, which indicates that the length that the polymer travels is a governing criterion for polymer reinjection operations. After production of the polymer, the polymer must be analyzed for any degradation and the extent to which the polymer degraded must be quantified. Based on the intended application of the polymer, it can therefore be assessed whether or not the polymer can be reinjected. 

## 4. Conclusions

This research investigates some of the parameters that may impact polymer degradation during injection and propagation through the rock for the purpose of enhanced oil recovery. Based on the obtained results, the ability to reuse the injected polymer through reinjection in terms of cost reduction and material sustainability was assessed. The main conclusions obtained from this research are as follows:

- Polymer degradation is strongly influenced by factors such as the pore size and the design injection length; therefore, it must be designed based on these parameters.
- Optimization of the polymer weight percent is an important design consideration to reduce cost and avoid excessive polymer degradation.
- Polymer degradation is predominantly an irreversible process which indicates that if the polymer is structurally degraded, it has to be disposed which results in material loss and operational costs.
- Excessive polymer hydrolysis can result from high injection pressures and high shearing which can generate high temperatures; thus, polymer hydrolysis is not only caused by reservoir temperature.
- Polymer degradation decreases significantly with an increase in the pore size of the porous media, which indicates that the polymer injected into the fractures due to the high fracture widths is less susceptible to degradation compared to the polymer injected into the formation matrix which usually has much smaller pores.

## Data Availability

The datasets generated during and/or analyzed during the current study are available from the corresponding author upon reasonable request.

## References

[1] Baushin V., Llc R.I., Muslimov R., Nikiforov A., Ramazanov R. (2023). On oil recovery of fractured-porous formations during cyclic and polymer-cyclic waterflooding (Russian). Neft. Khoz..

[2] Guo H., Wang Z., Dang S., Wen R., Lyu X., Liu H., Yang M. What is Learned from Polymer Flooding Practices in Offshore Reservoirs?. Proceedings of the Offshore Technology Conference.

[3] Kruglov D.S., Kornilov A.V., Tkachev I.V., Altynbaeva D.R., Sansiev G.V., Fedorchenko G.D., Fursov G.A., Ponomarenko D.M. (2023). Development of surfactant-polymer flooding technology for carbonate reservoirs with high salinity formation water and high reservoir temperature. Neft. Khoz..

[4] Khlaifat A.L., Dakhlallah D., Sufyan F. (2022). A Critical Review of Alkaline Flooding: Mechanism, Hybrid Flooding Methods, Laboratory Work, Pilot Projects, and Field Applications. Energies.

[5] Khorsandi S., Qiao C., Johns R.T. (2016). Displacement Efficiency for Low-Salinity Polymer Flooding Including Wettability Alteration. SPE J..

[6] Hamouda A.A., Chughtai S. (2018). Miscible CO_2_ Flooding for EOR in the Presence of Natural Gas Components in Displacing and Displaced Fluids. Energies.

[7] Fakher S., Imqam A. (2020). A data analysis of immiscible carbon dioxide injection applications for enhanced oil recovery based on an updated database. SN Appl. Sci..

[8] Fakher S., Abdelaal H., Elgahawy Y., Imqam A. (2019). A characterization of different alkali chemical agents for alkaline flooding enhanced oil recovery operations: An experimental investigation. SN Appl. Sci..

[9] Zhang F., Jiang Y., Liu P., Wang B., Sun S., Hua D., Zhao J. (2022). Laboratory Experimental Study on Polymer Flooding in High-Temperature and High-Salinity Heavy Oil Reservoir. Appl. Sci..

[10] Seright R.S., Wang D., Lerner N., Nguyen A., Sabid J., Tochor R. (2018). Can 25-cp Polymer Solution Efficiently Displace 1,600-cp Oil During Polymer Flooding?. SPE J..

[11] Seright R., Zhang G., Akanni O.O., Wang D. (2012). A Comparison of Polymer Flooding with In-Depth Profile Modification. J. Can. Pet. Technol..

[12] Sheng J., Leonhardt B., Al Azri N.S. (2015). Status of Polymer-Flooding Technology. J. Can. Pet. Technol..

[13] Seright R.S. (2017). How Much Polymer Should Be Injected During a Polymer Flood? Review of Previous and Current Practices. SPE J..

[14] Fakher S. What are the Dominant Flow Regimes During Carbon Dioxide Propagation in Shale Reservoirs’ Matrix, Natural Fractures and Hydraulic Fractures?. Proceedings of the SPE Western Regional Meeting.

[15] Baijal S.K., Kumar S. (1976). Interaction During Polymer Flooding. SPE J..

[16] Clarke A., Howe A.M., Mitchell J., Staniland J., Hawkes L.A. (2016). How Viscoelastic-Polymer Flooding Enhances Displacement Efficiency. SPE J..

[17] Delamaide E., Zaitoun A., Renard G., Tabary R. (2014). Pelican Lake Field: First Successful Application of Polymer Flooding in a Heavy-Oil Reservoir. SPE Reserv. Evaluation Eng..

[18] Erincik M.Z., Qi P., Balhoff M.T., Pope G.A. (2018). New Method to Reduce Residual Oil Saturation by Polymer Flooding. SPE J..

[19] Smith F.W. (1970). The Behavior of Partially Hydrolyzed Polyacrylamide Solutions in Porous Media. J. Pet. Technol..

[20] Zhou Y., Muggeridge A.H., Berg C.F., King P.R. (2019). Effect of Layering on Incremental Oil Recovery from Tertiary Polymer Flooding. SPE Reserv. Evaluation Eng..

[21] Alsofi A.M., Blunt M.J. (2010). Streamline-Based Simulation of Non-Newtonian Polymer Flooding. SPE J..

[22] Bondor P.L., Hirasaki G.J., Tham M.J. (1972). Mathematical Simulation of Polymer Flooding in Complex Reservoirs. SPE J..

[23] Slater G., Ali S.F. (1970). Simulation of Oil Recovery by Polymer Flooding. J. Can. Pet. Technol..

[24] Tagavifar M., Fortenberry R., de Rouffignac E., Sepehrnoori K., Pope G.A. (2016). Heavy-Oil Recovery by Combined Hot Water and Alkali/Cosolvent/Polymer Flooding. SPE J..

[25] Wu Y., Dong M., Shirif E.E. (2011). Study of Alkaline/Polymer Flooding for Heavy-Oil Recovery Using Channeled Sandpacks. SPE Reserv. Eval. Eng..

[26] Druetta P., Raffa P., Picchioni F. (2019). Chemical enhanced oil recovery and the role of chemical product design. Appl. Energy.

[27] Zhao Q., Sun J., Lin Y., Zhou Q. (2010). Study of the properties of hydrolyzed polyacrylamide hydrogels with various pore structures and rapid pH-sensitivities. React. Funct. Polym..

[28] Luo J., Hou Z., Feng G., Liao J., Haris M., Xiong Y. (2022). Effect of Reservoir Heterogeneity on CO_2_ Flooding in Tight Oil Reservoirs. Energies.

[29] Hincapie R.E., Borovina A., Clemens T., Hoffmann E., Tahir M., Nurmi L., Hanski S., Wegner J., Janczak A. (2022). Optimizing Polymer Costs and Efficiency in Alkali–Polymer Oilfield Applications. Polymers.

[30] Kazempour M., Sundstrom E.A., Alvarado V. (2012). Effect of Alkalinity on Oil Recovery During Polymer Floods in Sandstone. SPE Reserv. Evaluation Eng..

[31] Fakher S., El-Tonbary A., Abdelaal H., Elgahawy Y., Imqam A. Increasing Oil Recovery from Unconventional Shale Reservoirs Using Cyclic Carbon Dioxide Injection. Proceedings of the SPE Europec.

[32] Koh H., Lee V.B., Pope G.A. (2017). Experimental Investigation of the Effect of Polymers on Residual Oil Saturation. SPE J..

[33] Novosad J., Ionescu-Forniciov E., Mannhardt K. (1984). Polymer Flooding in Stratified Cores. Pet. Soc. Can..

[34] Fakher S., El-Tonbary A., Abdelaal H., Elgahawy Y., Imqam A. Carbon Dioxide Sequestration in Unconventional Shale Reservoirs Via Physical Adsorption: An Experimental Investigation. Proceedings of the SPE Europec.

[35] Seright R.S., Seheult J.M., Talashek T. Injectivity Characteristics of EOR Polymers. Proceedings of the SPE Annual Technical Conference and Exhibition.

[36] Seright R. (2010). Potential for Polymer Flooding Reservoirs with Viscous Oils. SPE Reserv. Eval. Eng..

